# Protective Effect of Thalidomide on 2,4,6-Trinitrobenzenesulfonic Acid-Induced Experimental Colitis in Rats via the Inhibition of T Helper 17 Cells

**DOI:** 10.1155/2020/8861854

**Published:** 2020-07-20

**Authors:** Ying Xie, Dandan Li, Xiaoshuang Luan, Shuang Jin, Bo Yan, Feng Tian

**Affiliations:** ^1^Department of Gastroenterology, Shengjing Hospital of China Medical University, Shenyang 110004, China; ^2^Department of Gastroenterology, General Hospital of Fushun Mining Bureau, Fushun 113000, China; ^3^Department of Gastroenterology, Dandong First Hospital, Dandong 118000, China; ^4^Department of Gastroenterology, The Third Affiliated Hospital of Qiqihar Medical University, Qiqihar 161000, China; ^5^Department of Gastroenterology, The People's Hospital of Liaoning Province, Shenyang 110000, China

## Abstract

**Objective:**

To observe the effects of thalidomide on 2,4,6-trinitrobenzenesulfonic acid- (TNBS-) induced experimental colitis in rats and to explore the possible mechanism of thalidomide in the treatment of CD.

**Methods:**

Forty SD rats were randomly assigned into a healthy control group and TNBS-induced colitis groups, including an untreated TNBS-induced colitis group, a low-dose thalidomide group, and a high-dose thalidomide group, with 10 rats in each. After 7 days, the disease activity index (DAI), colon macroscopic damage index (CMDI), and tissue damage index (TDI) were evaluated. The colonic protein and mRNA expression levels of interleukin-6 (IL-6), IL-17, IL-23, and retinoic acid receptor-related orphan nuclear receptor gamma t (ROR*γ*t) were determined using immunohistochemistry, western blot, and qRT-PCR.

**Results:**

Relative to the untreated TNBS-induced colitis group, the DAI, CMDI, and TDI were all reduced following the administration of thalidomide. Analytical testing (immunohistochemistry, western blot, and qRT-PCR) shows that IL-6, IL-17, IL-23, and ROR*γ*t protein and mRNA expression levels were significantly reduced by thalidomide (*p* < 0.05 for all) and that these levels were significantly lower in the high-dose thalidomide group than in the low-dose thalidomide group (*p* < 0.05 for all).

**Conclusions:**

Thalidomide effectively alleviated the symptoms and intestinal inflammatory injury induced by TNBS in rats, the effect of which was dose-dependent. The underlying mechanism may be a reduction in the expression levels of IL-6, IL-17, IL-23, and ROR*γ*t in colonic tissue and then subsequent inhibition of the differentiation and function of Th17 cells, thus further alleviating the intestinal inflammatory response.

## 1. Introduction

Inflammatory bowel disease (IBD) is a group of chronic, recurrent, and nonspecific inflammatory diseases that include two main entities: Crohn's disease (CD) and ulcerative colitis (UC). The etiology and pathogenesis of CD are not completely clear; however, the incidence of CD has been associated with multiple factors, based both on genetic susceptibility due to activation of antigen and immune responses in vivo and on environmental factors such as infection, diet interaction, and intestinal immune system imbalance, which cause intestinal mucosal damage and nonspecific intestinal inflammation.

The activation of T cells is a key to the induction of the intestinal immune response and subsequent inflammation. Naive CD4^+^T cells can differentiate into T helper 1 (Th1) cells, Th2 cells, Th17 cells, and T regulatory (Treg) cells under the action of different cytokines. CD is thought to be a Th1-mediated disease [1]. In recent years, increasing studies have shown that Th17 cells and its related cytokines are closely associated with the pathogenesis of CD. With respect to the differentiation of Th17 cells, TGF-*β* 1 and IL-6 are initiating factors, retinoic acid receptor-related orphan nuclear receptor gamma t (ROR*γ*t) is a key transcription factor, IL-23 is mainly involved in the maturation, amplification, and function maintenance of Th17 cells, and IL17 is its main effector [1–5]. Currently, exploration of the role of Th17 cells and their related cytokines in the pathogenesis of CD is a popular research topic.

At present, the main drugs for CD are 5-amino salicylic acid (5-ASA), glucocorticoids and immunosuppressants (azathioprine, cyclosporine A), and biological agents; however, these drugs have different limitations and side effects. For CD patients who are in the active stage, glucocorticoids can induce remission, but long-term use can lead to severe side effects such as osteoporosis, diabetes, and hypertension. Immunosuppressive agents such as azathioprine (AZA) can be used for CD reduction and maintenance of remission, but it takes a long time for the drug to take effect. The tumor necrosis factor alpha (TNF-*α*) antagonist, infliximab, is effective in the treatment of CD; however, the expensive cost limits its application.

Thalidomide was first developed in the late 1950s and was commonly prescribed to pregnant women. It was removed from the market due to a widespread outbreak of birth defects in Europe, particularly phocomelia, shortening of the long bones in the extremities, usually the upper arms. Until 1997, thalidomide was promoted for its therapeutic effect on IBD; however, Wettstein et al. [6] reported a case of CD treated with thalidomide, which was not effective in corticosteroid treatment. Thalidomide was used by physicians to treat a wide array of immune-mediated disorders.

Clinical research [7] has found that in children and adolescents with refractory CD, as compared with placebo, thalidomide resulted in improved clinical remission following 8 weeks of treatment and longer-term maintenance of remission in an open-label follow-up. Another clinical research study [8] reported that thalidomide reduces TNF-*α* and IL-12 production in patients with chronic active CD. Lazzerini et al. [9] showed that in a long-term analysis of data from two clinical trials involving pediatric patients with CD or UC, 52-week treatment with thalidomide led to clinical remission in 54.3% of patients with ileocolonic or colonic disease; of these patients, 75.3% displayed mucosal healing and 52.6% also displayed histological healing. Recent animal studies have demonstrated that thalidomide is effective in the management of CD-like TNBS-induced colitis in rats due to the suppression and downregulation of NF-*κ*B, TNF-*α*, IL-1*β*, IL-12, VEGF, endothelial adhesion molecules, and proinflammatory chemokines and upregulation of the anti-inflammatory cytokine IL-10 [10–12]. However, reports regarding the effects of thalidomide on Th17 cells function are lacking.

In the present study, the potential protective effects of thalidomide against intestinal inflammation were elucidated using a 2,4,6-trinitrobenzenesulfonic acid- (TNBS-) induced experimental colitis model in rats. Changes in Th17 cells-related cytokines and an associated transcription factor were measured during colitis induction to explore the possible mechanism of thalidomide in the treatment of CD.

## 2. Materials and Methods

### 2.1. Animals

A total of 40 Sprague Dawley rats (male, 20; female, 20), weighing 180–220 g, were purchased from Beijing HFK Bioscience Co., Ltd. (Beijing, China). The rats were fed in an SPF environment, at a temperature of 22 ± 2°C and 60% humidity, and were allowed to freely eat and drink. The animals were acclimated for seven days prior to the experiment. This study was approved by the Ethics Committee of Shengjing Hospital of China Medical University (No. 2015PS160K).

### 2.2. Reagents

Antibodies for immunohistochemistry and western blot were obtained as follows: IL-6, IL-17, IL-23, and ROR*γ*t (Abcam, Cambridge, MA, USA), GAPDH (Abcam, Cambridge, MA, USA), and Horseradish enzyme labeled goat anti-rabbit IgG antibody II (Proteintech, Rosemont, IL, USA). PrimeScript RT reagent kit and SYBR Premix Ex Taq (Tli RNaseH Plus) for qRT-PCR were purchased from Takara (Otsu, Japan). Primers were designed by Sangon Biotech (Shanghai) Co., Ltd. (Shanghai, China). 2,4,6-Trinitrobenzenesulfonic acid was obtained from SHYY-BIO (Shanghai, China) and thalidomide was obtained from Changzhou Pharmaceutical Factory. Co., Ltd. (Jiangsu, China).

### 2.3. 2,4,6-Trinitrobenzenesulfonic Acid- (TNBS-) Induced Colitis Model

The TNBS-induced colitis was induced as a previous method [13]. Rats were fasted for 24 hours with freely drinking and were randomly divided into a healthy control group and TNBS-induced colitis groups, including an untreated TNBS-induced colitis group, a low-dose thalidomide group, and a high-dose thalidomide group, with 10 rats in each. For the TNBS-induced colitis groups, rats were anesthetized by isoflurane and treated with TNBS (25 mg/kg, 50% ethanol solution) coloclysis. The rats in the healthy control group were treated with 0.9% NaCl solution coloclysis. Starting on the second day, thalidomide was administered intragastrically once daily (low-dose group, 100 mg/kg thalidomide and high-dose group, 200 mg/kg thalidomide) during the induction of colitis, but the rats in the healthy control group and the untreated TNBS-induced colitis group had received 400 *μ*L of 0.9% NaCl solution by the same procedure. All rats freely ate food and drank water. After the seven-day induction of colitis, rats were euthanized by isoflurane. Colonic tissue specimens were assessed macroscopically prior to being excised and fixed with 4% paraformaldehyde and were dehydrated and paraffin-embedded for histological analysis and immunohistochemistry. The remaining tissues were frozen at −80°C for western blot and qRT-PCR analysis.

### 2.4. Evaluation of the Disease Activity Index (DAI)

Under aseptic technique, 100 mg stool was collected for bacterial microflora analysis. DAI scores were assessed according to the previously established scoring system [14] (Table 1).

### 2.5. Evaluation of the Colon Macroscopic Damage Index (CMDI)

The CMDI was assessed according to the criteria described in Table 2 [15].

### 2.6. Evaluation of the Tissues Damage Index (TDI)

Tissues were stained with hematoxylin and eosin (HE). TDI was assessed using a modified version of the histopathological grading system described by MacPherson and Pfeiffer, as shown in Table 3 [16].

### 2.7. Immunohistochemistry

Tissues were deparaffinized and rehydrated and then were boiled in 0.01 M citrate buffer. Hydrogen peroxide (0.3%) was applied for 20 minutes, and normal goat serum was applied subsequently for 15 minutes. Sections were incubated overnight at 4°C within antibodies raised in rabbit against IL-6 (1 : 600), IL-17 (1 : 500), IL-23 (1 : 200), and ROR*γ*t (1 : 100). The sections were incubated with poly-HRP anti-rabbit IgG. PBS was used as a negative control. Sections were incubated with secondary antibody and then with horseradish peroxidase labeled streptomycin. Five fields of each section from each rat in each group were selected for image analysis. The average optical density was obtained to quantitate the expression levels of IL-6, IL-17, IL-23, and ROR*γ*t.

### 2.8. Quantitative Real-Time PCR (qRT-PCR)

Total RNA was extracted from colonic tissue using TRIzol, and cDNA was reverse-transcribed by PrimeScript RT reagent kit. PCR amplification system was prepared by using SYBR® Premier Ex Taq II. The two-step PCR amplification was carried out by using lightcycle480 II as follows: one 5 minutes at 95°C, followed by 15 seconds at 95°C and 30 seconds at 60°C, for 40 cycles. GAPDH was used as the internal reference. The primer sequences are listed in Table 4. The expression of mRNA was calculated by the 2^−ΔΔ^ct^^ method. Experiments were carried out in triplicate.

### 2.9. Western Blot

Colonic tissue was subjected to sodium dodecyl sulfate-polyacrylamide gel electrophoresis and transferred to PVDF membranes. Following blocking with 1% BSA, membranes were incubated overnight at 4°C with antibodies as follows: IL-6 (1 : 400), IL-17 (1 : 400), IL-23 (1 : 200), and ROR*γ*t (1 : 1000). The next day, the membranes were incubated with Horseradish enzyme labeled goat anti-rabbit IgG antibody II (1 : 5000) at room temperature for 2 hours. ECL was used for detection, and bands were quantitated by gel densitometry (Image J open-source software), using GAPDH as the loading control.

### 2.10. Statistical Analysis

Statistical analysis was performed by one-way analysis of variance (ANOVA) using the SPSS v22.0 software. Results are expressed as mean ± SD, and statistical significance was set at *p* < 0.05.

## 3. Results

### 3.1. DAI Scores

**Figure 1:**
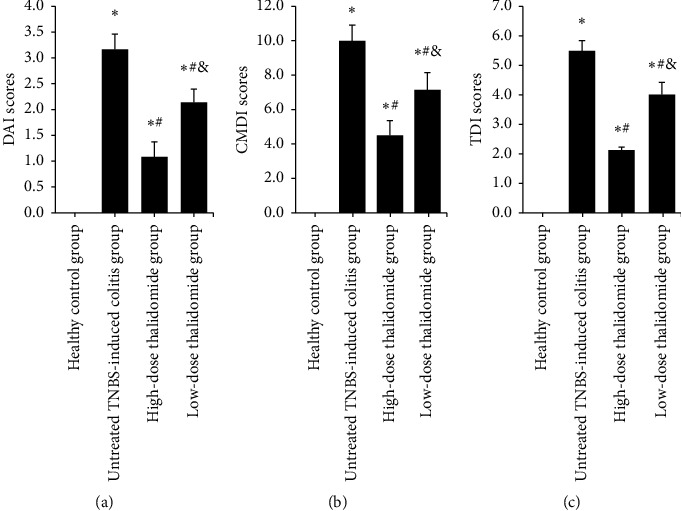
The DAI scores, CMDI scores, and TDI scores in each group. (a) DAI scores; (b) CMDI scores; and (c) TDI scores. Values are given as mean ± SD, *n* = 10. ^*∗*^*p* < 0.05 versus the healthy control group; ^#^*p* < 0.05 versus the untreated TNBS-induced colitis group; and ^&^*p* < 0.05 versus the high-dose thalidomide group.

Rats in the healthy control group generally showed the lowest DAI scores. In contrast, rats treated with TNBS had significantly higher DAI scores than those in the healthy control group (*p* < 0.05). The untreated TNBS-induced colitis group, which scored the highest, also differed significantly from the two thalidomide treated groups (*p* < 0.05). Compared with the low-dose thalidomide group, the high-dose thalidomide group had lower DAI scores (*p* < 0.05), suggesting a dose-dependent effect of thalidomide (Figure 1(a)).

### 3.2. CMDI Scores

All TNBS-induced colitis groups had significantly higher CMDI scores than those in the healthy control group (*p* < 0.05). In contrast to the untreated TNBS-induced colitis group, all thalidomide-treated groups showed lower CMDI scores (*p* < 0.05). There was a statistical difference (*p* < 0.05) between the low-dose thalidomide group and the high-dose thalidomide group, the latter of which had lower score, indicating a dose-dependent effect of thalidomide (Figure 1(b)).

### 3.3. HE Staining and TDI Scores

**Figure 2:**
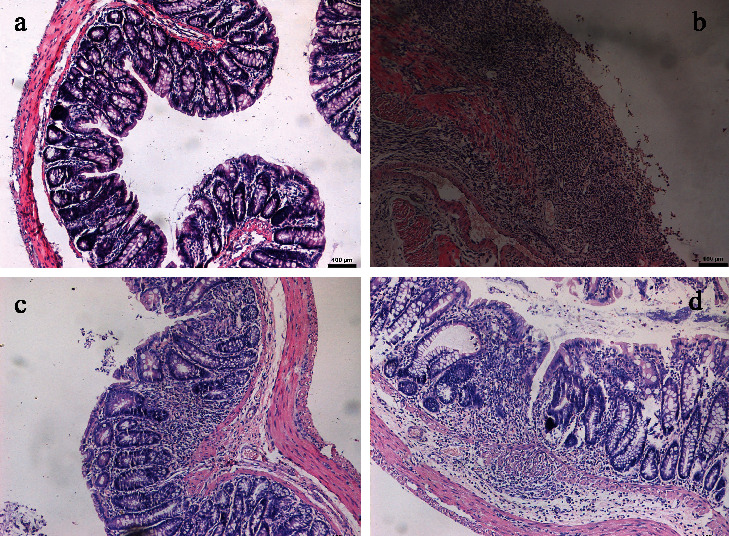
HE staining (×100). (a) Healthy control group; (b) untreated TNBS-induced colitis group; (c) high-dose thalidomide group; and (d) low-dose thalidomide group.

According to HE staining, the histopathology of the colonic tissue in the healthy control group was within normal limits; the glandular cells were arranged in order, with normal crypts without inflammatory cell infiltration. Microscopic examination showed lower levels of damage in rats treated with thalidomide, whereas in the untreated TNBS-induced colitis group, there was widespread destruction of the mucosa, with crypts and transmural infiltration of neutrophils, monocytes, and lymphocytes and defects in the epithelium (Figure 2). In each of the TNBS-induced colitis groups, the TDI scores were significantly higher than those in the healthy control group (*p* < 0.05). In the two thalidomide-treated groups, significantly decreased TDI scores were detected as compared with the untreated TNBS-induced colitis group (*p* < 0.05 for all), with the high-dose thalidomide group showing a lower score than the low-dose thalidomide group (*p* < 0.05) (Figures 1(c) and 2).

### 3.4. Immunohistochemistry

**Figure 3:**
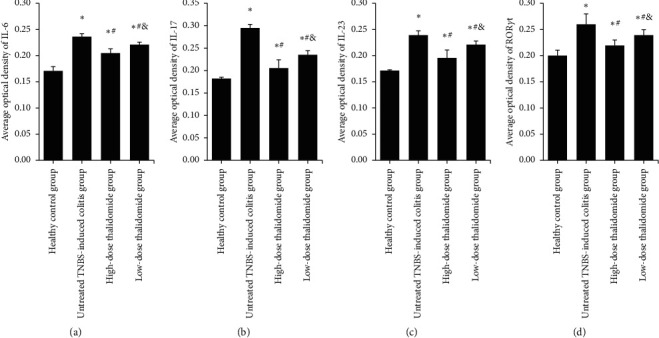
(a–d) Analysis of IL-6, IL-17, IL-23, and ROR*γ*t expression by average optical density analysis of immunohistochemistry images. Values are given as mean ± SD, *n* = 10. ^*∗*^*p* < 0.05 versus the healthy control group; ^#^*p* < 0.05 versus the untreated TNBS-induced colitis group; and ^&^*p* < 0.05 versus the high-dose thalidomide group.

**Figure 4:**
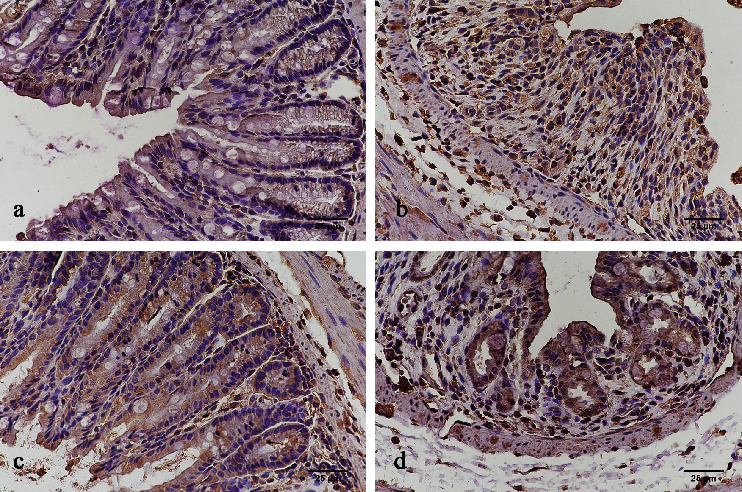
IL-6 expression in each group by immunohistochemistry (400x). (a) Healthy control group; (b) untreated TNBS-induced colitis group; (c) high-dose thalidomide group; and (d) low-dose thalidomide group.

**Figure 5:**
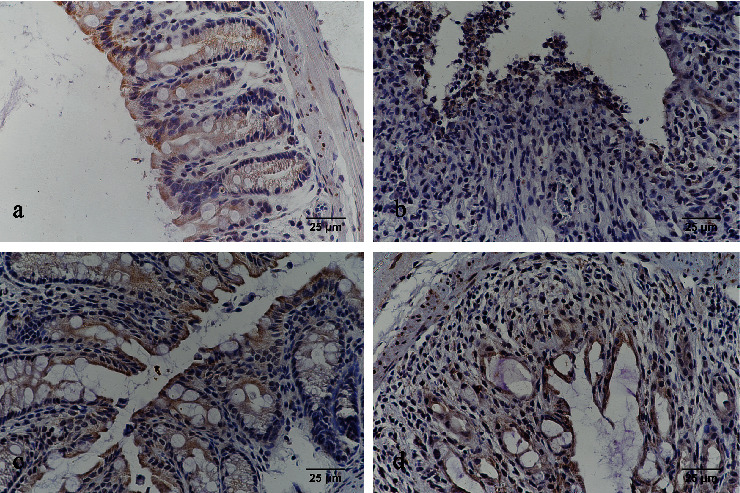
IL-17 expression in each group by immunohistochemistry (400x). (a) Healthy control group; (b) untreated TNBS-induced colitis group; (c) high-dose thalidomide group; and (d) low-dose thalidomide group.

**Figure 6:**
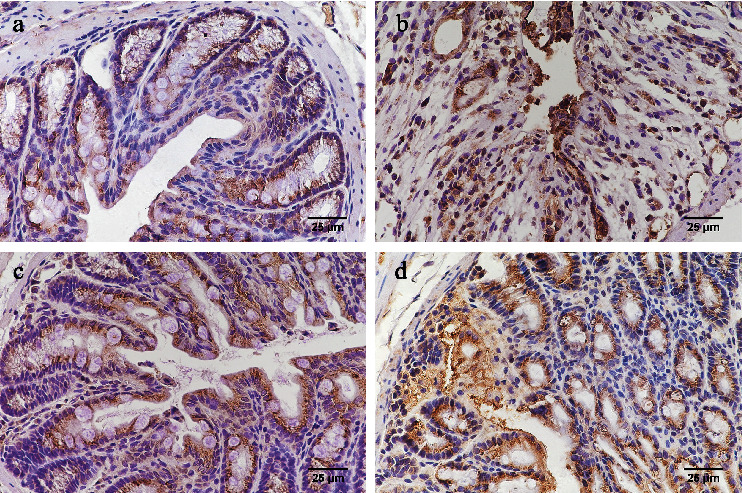
IL-23 expression in each group by immunohistochemistry (400x). (a) Healthy control group; (b) untreated TNBS-induced colitis group; (c) high-dose thalidomide group; and (d) low-dose thalidomide group.

**Figure 7:**
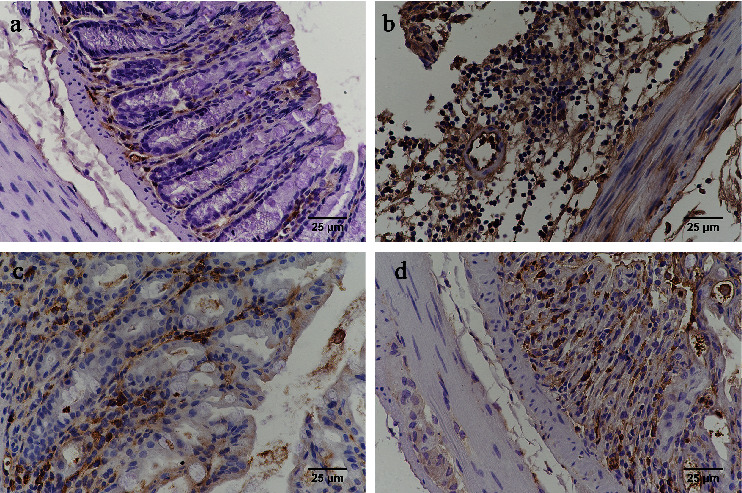
ROR*γ*t expression in each group by immunohistochemistry (400x). (a) Healthy control group; (b) untreated TNBS-induced colitis group; (c) high-dose thalidomide group; and (d) low-dose thalidomide group.

In the healthy control group, IL-6, IL-17, and IL-23 were expressed in the glandular epithelium, intestinal epithelium, inflammatory cells of the lamina propria, and the submucosa with brown granular distribution, whereas ROR*γ*t was seen only in the inflammatory cells of the lamina propria. For IL-6, IL-17, and IL-23, immunohistochemical staining was noticed in the cytoplasm, whereas ROR*γ*t staining was observed at the nucleus. Compared with the healthy control group, the expression levels of IL-6, IL-17, IL-23, and ROR*γ*t significantly increased after TNBS induction (*p* < 0.05 for all, Figures 3(a)–3(d)). The untreated TNBS-induced colitis group had the highest expression levels of IL-6, IL-17, IL-23, and ROR*γ*t as compared with the thalidomide-treated groups (*p* < 0.05 for all, Figures 3(a)–3(d)). Moreover, the high-dose thalidomide group had significantly less brown granular distribution than that in the low-dose thalidomide group (*p* < 0.05, Figures 3(a)–3(d)). The immunohistochemical staining patterns observed for IL-6, IL-17, IL-23, and ROR*γ*t were shown in Figures 4–7, respectively.

### 3.5. qRT-PCR

**Figure 8:**
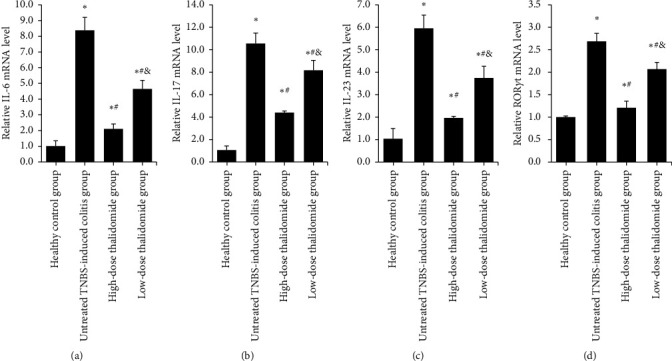
(a–d) Analysis of IL-6, IL17, IL23, and ROR*γ*t mRNA expression by qRT-PCR. Values are given as mean ± SD, *n* = 10. ^*∗*^*p* < 0.05 versus the healthy control group; ^#^*p* < 0.05 versus the untreated TNBS-induced colitis group; and ^&^*p* < 0.05 versus the high-dose thalidomide group.

IL-6, IL-17, IL-23, and ROR*γ*t were determined by qRT-PCR, and significant differences were observed in the expression levels of all these mRNA when comparing the TNBS-induced colitis groups with the healthy control group, while the addition of thalidomide downregulated their mRNA expression (*p* < 0.05, as compared with the untreated TNBS-induced colitis group, Figure 8). Meanwhile, the high dose of thalidomide resulted in significantly lower mRNA expression levels of IL-6, IL-17, IL-23, and ROR*γ*t than the low dose of thalidomide (*p* < 0.05 for all, Figure 8).

### 3.6. Western Blot

**Figure 9:**
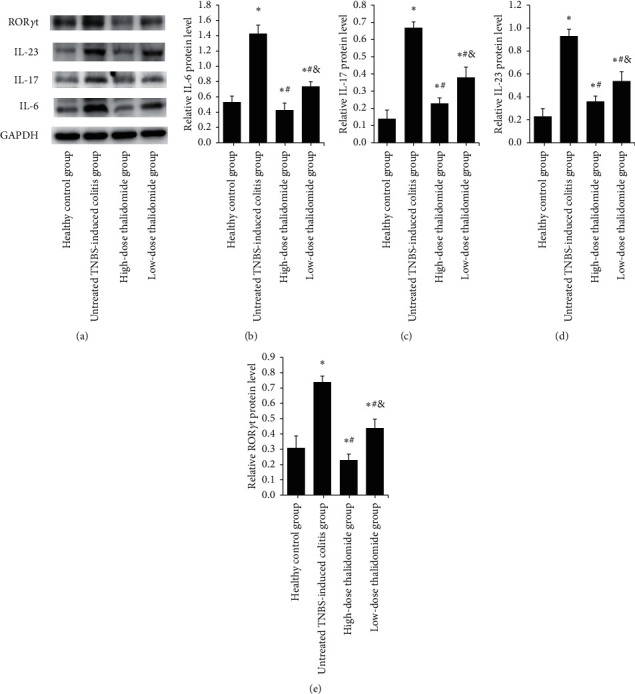
(a) IL-6, IL17, IL23, and ROR*γ*t expression in each group as seen by western blot; (b–e) analysis of IL-6, IL17, IL23, and ROR*γ*t protein levels by western blot. Values are given as mean ± SD, *n* = 10. ^*∗*^*p* < 0.05 versus the healthy control group; ^#^*p* < 0.05 versus the untreated TNBS-induced colitis group; and ^&^*p* < 0.05 versus the high-dose thalidomide group.

In the healthy control group, IL-6, IL-17, IL-23, and ROR*γ*t expression levels were low; however, instillation of TNBS significantly upregulated their expression (*p* < 0.05, as compared with the healthy control group), while the addition of thalidomide downregulated their expression (*p* < 0.05, as compared with the untreated TNBS-induced colitis group). Meanwhile, the high dose of thalidomide resulted in significantly lower expression levels of IL-6, IL-17, IL-23, and ROR*γ*t than the low dose of thalidomide (*p* < 0.05) (Figure 9).

## 4. Discussion

IBD is a chronic immune-mediated intestinal condition. UC and CD are the two major types of IBD. CD is a chronic granulomatous inflammation that can involve any part of the alimentary tract, typically the terminal ileum and adjacent colon. The pathology of CD often manifests as transmural inflammation, with multiple segmental and asymmetrical distribution. Symptoms include abdominal pain and diarrhea, which is often complicated by intestinal perianal fistulization or obstruction. The etiology and pathogenesis of CD are not completely clear; nevertheless, at present, it is believed to be based on the genetic susceptibility to interactions with environmental factors, diet, and infection, causing an imbalance of the intestinal immune system and nonspecific gastrointestinal inflammation.

Currently, the main drugs for the treatment of CD include 5-ASA, steroid hormones, immune-suppressants (azathioprine, cyclosporin A), and biological agents; however, these drugs have different limitations and side effects. Thalidomide, as an immune-modulator, has been widely used in the treatment of autoimmune diseases. In recent years, thalidomide has also been used in the treatment of IBD. In 1979, Waters et al. [17] reported the first case of UC successfully treated with thalidomide and until 1997, thalidomide was promoted for its therapeutic effect on IBD as a result of a study by Wettstein et al. [6]. In subsequent clinical studies, thalidomide has been found to play a role in the induction and maintenance of CD remission. The results of a 2013 randomized controlled trials (RCTs) [7] study of thalidomide for the treatment of refractory CD in children show that the eighth week remission rate of the thalidomide-treated group was 46.4% as compared with 11.5% in the placebo group, and the difference was statistically significant. A 2016 retrospective multicenter study found that thalidomide had a 12-month clinical remission rate of 54% for adults with refractory CD [18]. In 2014, Consensus Guidelines of the ECCO/ESPGHAN regarding the medical management of pediatric CD suggested that thalidomide could be used in the treatment of CD patients who were unresponsive or resistant to TNF-*α* [19].

At present, the underlying mechanisms of thalidomide in the treatment of CD are understood to be mainly immune-regulatory, anti-inflammatory, antiangiogenesis, and intestinal mucosal barrier function effects. Kim et al. [20] found that thalidomide can inhibit the expression of TNF-*α* and NF-*κ*B, and other studies [21] have shown that thalidomide can inhibit angiogenesis by reducing the proliferation of VEGF and human intestinal microvascular endothelial cells (HIMEC). Further research [22] has shown that thalidomide can reduce the levels of TNF-*α*, IL-1, IL-6, MPO, and NO in TNBS-induced mouse colitis, in turn reducing the inflammatory response in CD.

To further explore the mechanism of thalidomide in the treatment of IBD, we established an experimental colitis model in rats using the TNBS/ethanol method. In 1984, the TNBS-induced colitis animal model was first successfully produced by Morris et al. [13], which is now the most studied cellular immune model used to reflect the pathological changes and drug efficacy in CD. Administration of the hapten 2,4,6-trinitrobenzenesulfonic acid, in 0.25 mL 50% ethanol as the “barrier breaker,” produced dose-dependent colonic ulceration and inflammation. The characteristics and relatively long duration of the inflammation and ulceration induced in this model afford an opportunity to study the pathophysiology of colonic inflammatory disease in a specifically controlled fashion. The results of this study have shown that compared with the healthy control group, the untreated TNBS-induced colitis group was significantly lower in mental state, food intake, activity degree, and weight (*p* < 0.05), and the DAI score was significantly increased (*p* < 0.05). The intestinal wall of the rats in the untreated TNBS-induced colitis group showed different degrees of edema, erosion, ulcers, and even necrosis, adhesion, and stenosis of the intestine. The CMDI score of the rats in the untreated TNBS-induced colitis group was significantly higher than that in the healthy control group (*p* < 0.05). Microscopy shows colonic epithelial necrosis and shedding of the colonic mucosa in the untreated TNBS-induced colitis group; moreover, a large number of infiltrated inflammatory cells, serious destruction of the glands, an increased number of ulcers, and lamina propria involvement were seen, with fibrosis of the granulation tissue present in a few cases. Furthermore, the TDI score was significantly higher in the TNBS-induced colitis groups than that in the healthy control group (all *p* < 0.05). These results indicate that we successfully established an experimental colitis model in rats. We used two different doses of thalidomide to intervene in the TNBS-induced colitis rats. The results show that compared with the rats in the untreated TNBS-induced colitis group, the general condition of the rats in the thalidomide-treated groups improved significantly, the weight loss was slower, and the DAI, CMDI, and TDI scores were significantly decreased (all *p* < 0.05). Microscopy has shown that the intestinal mucosa was improved by varying degrees, the infiltration of mucous and submucosal inflammatory cells was reduced, the structure of the glands was improved, the congestion and edema were reduced, the marginal epithelial cells of the hyperplasia were covered by the ulcer surfaces, and the granulation tissue had formed fibroses. In the high-dose thalidomide group, the general condition and pathological changes of the rats were significantly relieved as compared with those in the low-dose thalidomide group (*p* < 0.05), which indicates that thalidomide dose-dependently alleviated colitis injury in TNBS-induced colitis rats.

In recent years, increasing evidence has shown that Th17 cells are closely related to the pathogenesis of IBD and have become the research focus of IBD pathogenesis. Th17 cells are a relatively new subgroup of CD4^+^T cells, which once activated secrete cytokines such as IL-17A, IL-17F, IL-21, and IL-22, among which IL-17 is the main effector. IL-17 is a proinflammatory factor with a powerful ability to recruit and activate neutrophils. In addition, it can also stimulate fibroblasts, macrophages, and epithelial cells to produce a variety of proinflammatory mediators such as IL-1, IL-6, TNF-*α*, MCP-1, and MMPs. A previous study [23] showed that in Stat-6 (–/–) T-bet (–/–) mice, which are unable to generate Th1 or Th2 cells, IL-6 alone was sufficient to induce robust differentiation of Th17 cells; however, when activated by TGF-*β* alone, CD4^+^T cells differentiated into Foxp3^+^ Treg cells. ROR*γ*t is a key transcription factor controlling Th17 cell differentiation. IL-23 cannot induce the differentiation of T cells to Th17 cells; however, it can increase the expression of IL-6, IL-1*β*, and TNF-*α* by positive feedback and participate in the amplification and survival of Th17 cells.

In the past, the pathogenesis of CD was associated with the Th1-mediated immune response; however, in recent years, increasing studies have shown that Th17 cells and their related cytokines are closely associated with the pathogenesis of CD. Jiang et al. [24] reported that the expression of Th17 cells and their secreted cytokines (IL-17, IL-21, and IL-22) were increased in the intestinal mucosa of active IBD patients. Kobayashi et al. [25] found that CD4^+^ cells and IL-17 production were high in the lamina propria of mucosal samples from IBD patients who had undergone surgical resection. IL-23R and RORC were upregulated, indicating that IL-23 may play important roles in controlling the differential Th1/Th17 balance in both UC and CD, although Th17 cells may exist in both diseases. Another study [26] showed that IL-17 can interact with the submucosal fibromyocytes of the intestinal mucosa and upregulate the expression of IL-1, TNF-*α*, and IL-6 by activating the classical MAPK/NF-*κ*B signaling pathway to cause intestinal inflammation. IL-17RA (–/–) mice have been shown to be less susceptible to acute intestinal mucosal injury [27], and IL-17 (–/–) or the application of an anti-IL-17 antibody can alleviate TNBS induced experimental colitis in mice [28]. A clinical IBD study [29] showed that the clinical response and remission rates in the treatment group following administration of an anti-IL-6 antibody (PF-04236921) were better than those of in placebo group. IL-23 and IL-12 share p40 subunits, and several clinical studies [30–32] have confirmed that an anti-IL-12p40 monoclonal antibody is effective in the treatment of active CD patients.

To further investigate whether the underlying mechanism of thalidomide in the treatment of CD involves the modulation of Th17 cells, our experiment examined the levels of related cytokines and an associated transcription factor that affect the differentiation, amplification, and function of Th17 cells. We used immunohistochemistry, western blot, and qRT-PCR to evaluate the protein and mRNA expression levels of IL-23, IL-17, and IL-6 in the colonic tissue of rats. Statistical analysis results show that the protein and mRNA expression levels of IL-23, IL-17, and IL-6 in the untreated TNBS-induced colitis group were significantly higher than those in the healthy control and thalidomide-treated groups (*p* < 0.05). The protein and mRNA expression levels of IL-23, IL-17, and IL-6 in the high-dose thalidomide group were lower than those in the low-dose thalidomide group, showing a dose-dependent effect (*p* < 0.05). These results indicated that the protein and mRNA expression levels of IL-23, IL-17, and IL-6 were increased in colonic tissue of TNBS-induced colitis rats. Administration of thalidomide significantly dose-dependently reduced the protein and mRNA expression levels of IL-23, IL-17, and IL-6 in the colonic tissue of TNBS-induced colitis rats. Immunohistochemistry result shows that compared with the healthy control group, significantly increased expression of ROR*γ*t was observed following TNBS instillation (*p* < 0.05). The untreated TNBS-induced colitis group had a significantly higher expression level of ROR*γ*t as compared with thalidomide-treated groups (*p* < 0.05). Moreover, the high-dose thalidomide group had significantly less brown granular distribution than the low-dose thalidomide group (*p* < 0.05). According to western blot and qRT-PCR results, the expression of ROR*γ*t protein and mRNA in the colonic tissue of the untreated TNBS-induced colitis group was significantly higher than that in the healthy control group (*p* < 0.05). Furthermore, the ROR*γ*t protein and mRNA expression were significantly lower following the intervention of two different doses of thalidomide (all *p* < 0.05), with the high-dose thalidomide group showing a lower expression level than the low-dose thalidomide group (*p* < 0.05). Therefore, the present results indicate that thalidomide dose-dependently reduced the differentiation of Th17 cells by reducing the protein and mRNA expression of ROR*γ*t, slowing the progression of CD inflammation and promoting the healing of the mucous membrane.

## 5. Conclusions

In summary, our experimental results show that thalidomide can dose-dependently reduce the DAI, CMDI, and TDI scores of TNBS-induced colitis in rats, possibly via downregulation of the expression of the cytokines IL-6, IL-17, and IL-23, and the transcription factor, ROR*γ*t, inhibiting the differentiation and power of Th17 cells and alleviating the inflammatory reaction in the intestinal tract. The present study only shows that thalidomide affects the differentiation and function of Th17 cells; however, its underlying mechanism requires further study. There is a relationship between Th17 and Tregs; thus, exploration of the related mechanisms of Tregs is needed to evaluate the therapeutic effect of thalidomide and to provide a more sufficient theoretical basis for thalidomide in the treatment of CD.

In conclusion, thalidomide can effectively alleviate the symptoms and intestinal inflammatory injury induced by TNBS in rats, the effect of which is dose-dependent. The mechanism may be related to a reduction in the expression of IL-23, IL-17, IL-6, and ROR*γ*t in colonic tissue, followed by inhibition of the differentiation and function of Th17 cells, thus further alleviating the intestinal inflammatory response.

## Figures and Tables

**Table 1:** DAI criteria.

Grade	Weight loss (%)	Stool consistency	Occult/gross bleeding

0	0	Normal	N/A
1	1–5	Mild soft	—
2	5–10	Soft and wet	Hemoccult positive
3	10–20	Half-loose stool	—
4	>20	Loose stool	Gross bleeding

**Table 2:** CMDI criteria.

Score	Criteria

0	No damage
1	Hyperemia
2	Hyperemia and thickening without ulceration
3	Ulceration at a single site
4	Two or more sites of ulceration or inflammation
5	Ulceration or inflammation extending >1 cm along the length of colon
6–10	Damage covering >2 cm along the length of colon, with the score being increased by 1 for each additional centimeter of involvement

**Table 3:** TDI criteria.

Grade of colitis	Microscopic findings

0	No damage
I	Mild mucosal and/or submucosal inflammatory infiltrate and edema: punctuate mucosal erosions often associated with capillary proliferation, muscularis mucosa intact
II	Grade I changes involving 50% of the specimen
III	Prominent inflammatory infiltrate and edema frequently with deeper areas of ulceration extending through the muscularis mucosa into the submucosa, rare inflammatory cells invading the muscularis propria but without muscle necrosis
IV	Grade III changes involving 50% of the specimen
V	Extensive ulceration with coagulative necrosis bordered inferiorly by numerous neutrophils and fewer mononuclear cells; necrosis extends deeply into the muscularis propria
VI	Grade V changes involving 50% of the specimen

**Table 4:** PCR primer sequence.

Primer sets	Primer sequence

IL-6	L: 5′CACTTCACAAGTCGGAGGCT3′
	R: 5′TCTGACAGTGCATCATCGCT3′
IL-17	L: 5′CCATCCATGTGCCTGATGCT3′
	R: 5′AAGTTATTGGCCTCGGCGTT3′
IL-23	L: 5′ATAAGCACCTGCTGGACTCG3′
	R: 5′GGAACGGAGAAGAGAACGCT3′
ROR*γ*t	L: 5′CGACTTTTCCCACTTCCTCA3′
	R: 5′GCAGATGCTCCACTCTCCTC3′
GAPDH	L: 5′ATGTTTGTGATGGGTGTGAA3′
	R: 5′ATGCCAAAGTTGTCATGGAT3′

## Data Availability

The immunohistochemistry, western blot, and qRT-PCR data used to support the findings of this study are available from the corresponding author upon request via e-mail (tfdoc1088@163.com).
